# Altererythrobacter chaiwopuensis sp. nov., a novel alphaproteobacterial strain isolated from sediment of the Chaiwopu Lake

**DOI:** 10.1099/ijsem.0.007250

**Published:** 2026-08-10

**Authors:** Xin-Yao Li, Yi-Fei Yang, Yi-Wen Liang, Ze-Xian Wu, Xu-Rui Li, Shuai Li, Yong-Hong Liu, Lei Gao, Ting-Ting She, Bao-Zhu Fang, Wen-Jun Li

**Affiliations:** 1 State Key Laboratory of Ecological Safety and Sustainable Development in Arid Lands, China-Tajikistan Belt and Road Joint Laboratory on Biodiversity Conservation and Sustainable Use, Xinjiang Institute of Ecology and Geography, Chinese Academy of Sciences, Urumqi, 830011, PR China; 2 University of Chinese Academy of Sciences, Beijing 100049, PR China; 3 School of Biology and Food Engineering, Guangdong University of Education, Guangzhou, 510303, Guangdong, PR China; 4 Xinjiang Key Laboratory of Biodiversity Conservation and Application in Arid Lands, Xinjiang Institute of Ecology and Geography, Chinese Academy of Sciences, Urumqi, 830011, PR China; 5 State Key Laboratory of Biocontrol, Guangdong Provincial Key Laboratory of Plant Stress Biology, School of Life Sciences, Sun Yat-Sen University, Guangzhou, 510275, Guangdong, PR China

**Keywords:** *Altererythrobacter chaiwopuensis *sp. nov, chaiwopu lake, *Erythrobacteraceae*, polyphasic taxonomy

## Abstract

Two alphaproteobacterial strains, designated EGI L300112^T^ and EGI L300120, were isolated from sediment collected from Chaiwopu Lake in Xinjiang, China. The taxonomic position of the two strains was determined using polyphasic taxonomic analysis and phylogenomic analysis. Based on 16S rRNA gene sequence similarities, strain EGI L300112^T^ and EGI L300120 were both closely related to *Pelagerythrobacter rhizovicinus* AY-3R^T^ and shared the highest sequence identities of 96.2% and 96.3%, respectively. However, phylogenetic analysis based on the 16S rRNA gene and genomes clearly demonstrated that the two strains formed a distinct clade with validly published species of the genus *Altererythrobacter*. Furthermore, the two strains exhibited distinct phenotypic, physiological and genotypic characteristics that differentiate them from other related type species of the genus *Altererythrobacter* and related species in the family *Erythrobacteraceae*. Cells of the two strains were aerobic, Gram-stain negative, non-motile and rod-shaped. Optimal growth conditions for EGI L300112^T^ and EGI L300120 occurred on marine agar 2216 at pH 7 at 30 °C. The major respiratory quinone was Q-10. The detected polar lipids of two strains included diphosphatidylglycerol, phosphatidylglycerol, phosphatidylmethylethanolamine, phosphatidylcholine and unidentified phospholipids. The major fatty acids (>10%) were identified as summed feature 3 (C_16 : 1_*ω*7* c*/C_16 : 1_*ω*6 c) and summed feature 8 (C_18 : 1_*ω*7 c). The G+C content of strain EGI L300112^T^ and EGI L300120 was 61.2% and 61.4%, respectively. Based on differential phenotypic and genotypic characteristics of the two strains and related species in the genus *Altererythrobacter*, the two strains should be classified as representing a new species of this genus, for which the name *Altererythrobacter chaiwopuensis* sp. nov. is proposed. The type strain is EGI L300112^T^ (=MCCC 1K09432^T^=KCTC 8712^T^).

## Introduction

The genus *Altererythrobacter* of the family *Erythrobacteraceae*, order *Sphingomonadales*, class *Alphaproteobacteria* and phylum *Pseudomonadota*, was first proposed by Kae *et al.* in 2007 [1] and was reclassified by Xu *et al*. [2]. It currently comprises ten validly published species, including *Altererythrobacter aquiaggeris*, *Altererythrobacter arenosus*, *Altererythrobacter epoxidivorans*, *Altererythrobacter flavalbus*, *Altererythrobacter insulae*, *Altererythrobacter ishigakiensis*, *Altererythrobacter litoralis*, *Altererythrobacter lutimaris*, *Altererythrobacter rubellus* and *Altererythrobacter xiamenensis*. Members of this genus are Gram-stain-negative, aerobic and non-motile [2]. Strains belonging to the genus *Altererythrobacter* were isolated from various habitats, including river water [3], sand [4], marine sediment [5], tidal flat sediment [6], seawater [7], farmland soil [8] and red tide seawater [9]. Chemotaxonomically, species of *Altererythrobacter* typically contain ubiquinone Q-10 as the predominant respiratory quinone, and the major cellular fatty acid is usually summed feature 8 (C_18 : 1_* ω6*c and/or C_18 : 1_* ω7*c). Some members of this genus produce *β*-etherases with potential applications in lignin depolymerization [10] and the esterase Ali5 [11], which displays high resistance to metal ions and various organic solvents, and some *Altererythrobacter* strains can metabolize benzo[a]pyrene as the sole carbon and energy source [12].

Microorganisms in lake sediment play an important role in the process of the element cycle, humus decomposition and synthesis. To investigate the microbial diversity of lake sediment, we collected the lake sediment samples of Chaiwopu Lake, Xinjiang Uygur Autonomous Region, China. During a study of the diversity of cultivable micro-organisms in this lake sediment, two alphaproteobacterial strains designated as EGI L300112^T^ and EGI L300120 were isolated from the lake sediment samples. The analysis results based on 16S rRNA gene sequences indicated that the two strains belonged to the genus *Altererythrobacter* with a low sequence identity (≤97%). This study was conducted to determine the taxonomic status of the two novel strains with comparative analyses based on the phenotypic, chemotaxonomic and molecular characteristics between the two strains and related reference species in this genus.

## Methods

### Sample collection and bacterial isolation

The lake sediment samples were collected using sterile sampling devices. Samples were transported without temperature control and stored at 4 ℃. The samples were serially diluted in sterile water to 10^−4^ and a suspension (200 µl) was then spread onto marine agar 2216 plates [13]. The plates were incubated at 30 ℃ for 5 days. Single colonies were picked and purified by repeatedly streaking on the same medium. The purified strains EGI L300112^T^ and EGI L300120 were found to show low 16S rRNA gene sequence similarities compared to other validly published species and were chosen for detailed characterization later. Then, purified strains were stored in glycerol suspensions (20%, v/v) at −80 ℃.

### Morphological and physiological characterization

Gram staining was performed with a Gram stain kit (Solarbio), according to the manufacturer’s instructions. The morphological features of strains EGI L300112^T^ and EGI L300120 were observed by using transmission electron microscopy (JEM-1400FLASH, JEOL) after growing on the marine agar 2216 (Hopebio) at 30 ℃ for 3 days. Growth of the strains at different temperatures (4, 15, 20, 28, 30, 37, 45 and 50 ℃) and NaCl tolerance (0–8.0%, at intervals of 1.0 %, w/v) were tested on marine agar 2216 without NaCl for 7 days. The pH range (4.0–10.0, at intervals of 1.0 pH unit) for growth was determined on marine 2216 medium using the buffer system described by Xu *et al*. [14]. The presence of turbidity in a tube containing a semi-solid marine 2216 medium was utilized to assess the motility of cells [15]. Activities of catalase, oxidase and urease, hydrolysis of Tween (20, 40, 60 and 80), milk coagulation and peptonization, H_2_S production and starch hydrolysis were determined according to the conventional procedures as previously described by Fang *et al*. [16]. Substrate utilization of strains was tested using the Biolog GEN III (BIOLOGs) microplate system according to the manufacturer’s instructions. Other physiological and biochemical characteristics were determined using API ZYM (bioMérieux) and API 20 NE (bioMérieux) according to the manufacturer’s instructions.

### Chemotaxonomic characterization

Chemotaxonomic characterization of two strains was observed using several standard methods under similar conditions. The biomass for fatty acids was harvested from marine 2216 agar. According to the standard protocol of the Microbial Identification System (Sherlock version 6.1; MIDI database: TSBA6), the cellular fatty acids were extracted and determined. Menaquinones were extracted from lyophilized cells [17], and the extracts were purified and analysed by HPLC [18]. The polar lipids were extracted and separated by using two-dimensional TLC [19] based on the previous methods [20].

### Phylogenetic and genomic analyses

Genomic DNA was extracted and the 16S rRNA genes were amplified according to the method described by Xu *et al*. [21]. The 16S rRNA gene was amplified by using the universal primers 27F (5′-AGAGTTTGATCMTGGCTCAG-3′) and 1492R (5′-TACGGYTACCTTGTTACGAC-3′). The sequences were compared with available 16S rRNA gene sequences of the validly published species in the EzBioCloud server [21]. Phylogenetic analysis was conducted using mega 11 software [22] and subsequent to the multiple alignment of sequences performed by the clustal_w program [23]. The phylogenetic trees based on 16S rRNA gene sequences were constructed with neighbour-joining [24], maximum-likelihood [25] and maximum-parsimony [26] methods. The bootstrap based on 1,000 replications was employed to assess the reliability of each branch [27].

Whole-genome sequences of strains EGI L300112^T^ and EGI L300120 were performed by using the paired-end sequencing method with the Illumina platform at Novogene Biotech (Suzhou, PR China). Raw data were filtered using the quality-control software ‘sickle’ v1.33 before being assembled with SPAdes v3.15.5 with the parameters (--single -k 21, 33, 55, 77, 99) [28], and genome completeness and contamination of genomes of two strains were assessed utilizing CheckM [29]. The G+C contents were determined based on the genome sequences. The average nucleotide identity (ANI) and average amino acid sequence identity (AAI) of two strains with other related type species were computed with an online ANI calculator (https://www.ezbiocloud.net/tools/ani) [30] and JSpecies software [31], respectively. The digital DNA–DNA hybridization (dDDH) values between strains EGI L300112^T^ and EGI L300120 and closely related type strains were calculated using the Genome-to-Genome Distance Calculator 3.0 web server. For the phylogenomic analysis, GTDB-Tk v.2.3.0was used to create a multiple sequence alignment of 120 conserved bacterial marker genes. Then, IQ-TREE v.1.6.12 was employed to construct the phylogenetic tree [32], with bootstrap support values set at 1,000. The phylogenomic tree was visualized by the interactive Tree of Life (iTOL) [33]. Related genes of two strains were predicted using Prodigal v2.6.3 and annotated through DIAMOND v2.1.7.161 (*e*-value <1e-5, query coverage >60%) against KEGG (Kyoto Encyclopedia of Genes and Genomes) and COG (Clusters of Orthologous Groups of proteins) databases [34, 35].

## Results and discussion

### Phylogenetic analysis based on 16S rRNA gene

**Fig. 1.**
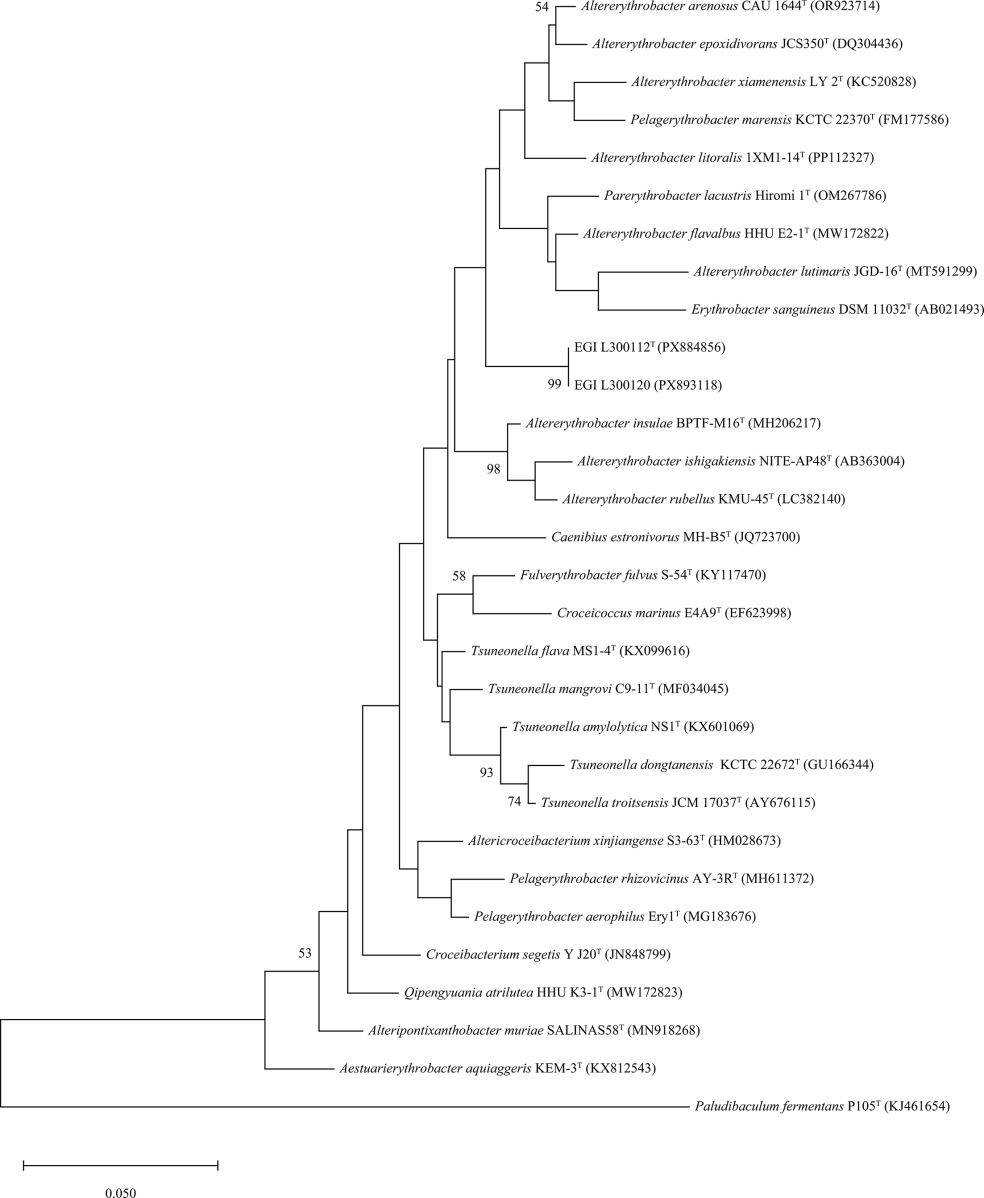
Maximum-likelihood phylogenetic tree based on 16S rRNA gene sequences showing the phylogenetic relationships between strains EGI L300112ᵀ and EGI L300120, the type strains of *Altererythrobacter* species and representatives of related taxa. Bootstrap values (>50%) based on 1,000 resamplings are shown at the branch nodes. *Paludibaculum fermentans* P105ᵀ (KJ461654) was used as the outgroup. Bar, 0.05 substitutions per nucleotide position.

The nearly complete 16S rRNA gene sequences of strain EGI L300112^T^ (1,501 bp) and EGI L300120 (1,501 bp) were deposited in GenBank under accession numbers PX884856 and PX893118, respectively. Analysis of sequences using the EzBioCloud server revealed that strains EGI L300112^T^ and EGI L300120 were closely related to *Pelagerythrobacter rhizovicinus* AY-3R^T^ (96.2% and 96.3%, sequence similarity), *Tsuneonella amylolytica* NS1^T^ (96.2% and 96.2%) and *Parerythrobacter lacustris* RS5-5^T^ (96.1% and 96.2%). Phylogenetic analysis based on 16S rRNA gene sequences using the maximum-likelihood algorithm showed that strains EGI L300112ᵀ and EGI L300120 formed a distinct clade, including validly published species of the genus *Altererythrobacter*, as well as *Pelagerythrobacter marensis* KCTC 22370ᵀ, *Parerythrobacter lacustris* Hiromi 1ᵀ and *Erythrobacter sanguineus* DSM 11032ᵀ, and was clearly separated from other members of the family *Erythrobacteraceae* (Fig. 1). The topological structure of the two strains with other related species in the neighbour-joining and maximum-parsimony trees was the same but different from the maximum-likelihood tree (Fig. S2). To further clarify the evolutionary relationships of the two strains, the genome-based topological analysis results could comprehensively elucidate their phylogenetic connections with the genus *Altererythrobacter* and other related genera.

### Genomic characteristics and phylogenomic analysis

**Fig. 2.**
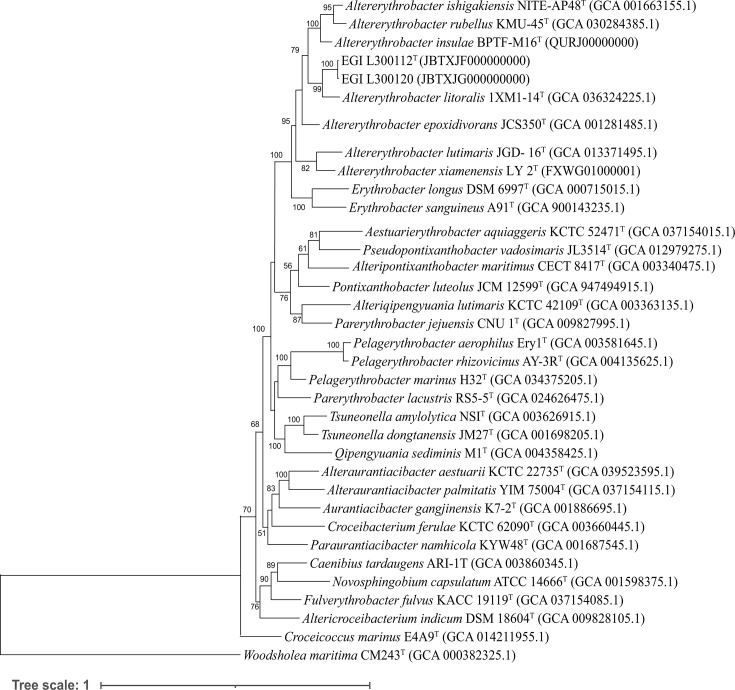
Phylogenomic tree reconstructed from 120 single-copy marker proteins showing the phylogenetic relationships of strains EGI L300112ᵀ and EGI L300120 with the type strains of *Altererythrobacter* species and representatives of related taxa. The two strains formed a distinct clade separate from those of the other described species with available genome sequences. Bootstrap values (≥50%) based on 1,000 replicates are shown at the branch nodes. *Woodsholea maritima* CM243ᵀ (GCA_000382325.1) was used as the outgroup.

The draft genome sequence of strain EGI L300112^T^ (GenBank accession JBTXJF000000000) was 2,682,049 bp in 25 scaffolds with N_50_ scaffold length of 1887,887, while the genome size of strain EGI L300120 (GenBank accession JBTXJG000000000) was 2,682,049 bp in 34 scaffolds, with N_50_ scaffold length of 2170,281. The completeness of the assembly genomes of strains EGI L300112^T^ and EGI L300120 was 99.5% and 99.4%, with contamination rates of 0.2% and 0.4%, respectively. The two strains had 2,579 and 2,586 protein-coding genes, respectively. The G+C content of the two strains was 61.2% and 61.4%, respectively. The ANI values between strains EGI L300112ᵀ and EGI L300120 and *A. litoralis* AY-3Rᵀ were 63.3% and 63.2%, respectively. The ANI values between the two strains and validly published species of the genus *Altererythrobacter* ranged from 37.5 to 63.3%, while the ANI value between the two strains themselves was 98.23%, which was above the generally species-level threshold (95–96%) (Fig. S3). To further clarify their taxonomic position, additional genome-based comparisons were performed with representatives of closely related genera within the family *Erythrobacteraceae*, including *Tsuneonella*, *Erythrobacter*, *Parerythrobacter* and *Croceicoccus*. Similarly, the AAI values between the two strains and related reference species of the genus *Altererythrobacter* ranged from 73.8 to 81.0% (Fig. S4). In addition, dDDH values between strains EGI L300112ᵀ and EGI L300120 and their closest relatives, including members of *Altererythrobacter* and other related genera, were all well below the 70% threshold for species delineation (Fig. S5). In all cases, ANI, AAI and dDDH values remained well below the species threshold, and the two strains represented one novel species of the genus *Altererythrobacter*. Furthermore, the phylogenomic tree based on the concatenated alignment of 120 marker genes showed that EGI L300112^T^ and EGI L300120 formed a distinct clade with other validly published species of the genus *Altererythrobacter*, and separated from other species of the family *Erythrobacteraceae* (Fig. 2).

COG categories distributions for the genes were presented in Table S1 (available in the online Supplementary Material). The most abundantly represented CDSs in the two strains were lipid transport and metabolism (I), translation, ribosomal structure and biogenesis (J), amino acid transport and metabolism (E), energy production and conversion (C), inorganic ion transport and metabolism (P) and posttranslational modification, protein turnover, chaperones (O). Two novel strains had a large number of CDS for inorganic ion transport and metabolism (P). This abundance may be because these strains were isolated from a saline environment, where their survival likely required the regulation of internal osmotic balance through the active transport of inorganic ions.

The distribution of KEGG functional pathways of the genes is outlined in Table S2. The most abundantly represented KEGG pathways in strains EGI L300112^T^ and EGI L300120 were carbohydrate metabolism, followed by amino acid metabolism, energy metabolism and metabolism of cofactors and vitamins. Specifically, according to the KEGG annotation results, strains EGI L300112^T^ and EGI L300120 possess complete pentose phosphate pathway (non-oxidative phase) and TCA cycle pathways. Moreover, the pentose phosphate pathway compensates for glycolysis (Embden–Meyerhof pathway), enabling its full functionality. KEGG annotation revealed that the genomes of strains EGI L300112ᵀ and EGI L300120 harbour the gene K00370 encoding a putative nitrate reductase alpha subunit. This genomic feature is consistent with the positive nitrate reduction phenotype observed in physiological tests, suggesting a potential capacity for nitrate reduction in these strains.

### Phenotypic characteristics

Cells of strains EGI L300112^T^ and EGI L300120 were Gram-stain-negative, non-motile and aerobic. The colonies were yellow, circular and convex with regular edges after 5 days of incubation on isolation medium at 28 ℃. Strain EGI L300112^T^ and EGI L300120 could grow on marine agar 2216, Reasoner’s 2A agar, Luria–Bertani agar, nutrient agar and tryptic soy agar, while no colonies were observed on other tested media at 28 ℃ for 15 days. Cells of strain EGI L300112^T^ and EGI L300120 were rod-shaped, with ~0.4–0.6 µm width and 0.9–1.5 µm length (Fig. S1). Strains EGI L300112^T^ and EGI L300120 were able to grow at 15–37 °C (optimum 28 °C), pH 6.0–8.0 (optimum pH 7.0) and in the presence of 0–7% (w/v) NaCl (optimum, 2 %, w/v). Strain EGI L300112^T^ and strain EGI L300120 were positive for catalase and hydrolysis of Tweens 20, 60 and 80. Strains EGI L300112^T^ and EGI L300120 were negative for oxidase and H_2_S generation. In the commercial kit API 20 NE, all strains exhibited positive results for glucosidase and galactosidase. For enzyme activity detected using API ZYM, the two strains tested positive for esterase, esterase lipase, leucine arylamidase, valine arylamidase, cystine arylamidase, trypsin, chymotrypsin, acid phosphatase, naphthol-AS-BI-phosphohydrolase and *α*-glucosidase. Differences in physiological characteristics between EGI L300112^T^, EGI L300120 and reference type species were presented in Table 1.

**Table 1.** Differentiating characteristics of strain EGI L300112^T^, EGI L300120 and their closely related type strains of the genus *Altererythrobacter* 1, EGI L300112^T^; 2, EGI L300120; 3. *A. litoralis* 1XM1-14^T^ [36]; 4, *P. rhizovicinus* Ery12^T^ [37]; 5, *Croceicoccus marinus* E4A9 ^T^ [38]; 6, *Tsuneonella dongtanensis* JM27 ^T^ [6]; 7, *Erythrobacter longus* Och01 ^T^ [39, 40].

Characteristic	1	2	3	4	5	6	7
Temperature range (℃)	15.0–37.0	15.0–37.0	10.0–35.0	10.0–42.0	4.0–42.0	10.0–37.0	20.0–45.0
pH range	6.0–8.0	6.0–8.0	5.0–9.0	5.0–9.5	6.0–9.0	6.0–10.0	6.0–9.0
NaCl range (%, w/v)	0–7.0	0–7.0	1.0–4.0	0–10.0	0–10.0	0–1.0	0–8.0
Colony colour	Yellow	Yellow	Yellow	Yellow	Yellow	Yellow	Orange
Cell size (width×length; μm)	0.4–0.6×0.9–1.5	0.4–0.6×0.9–1.5	0.4–0.6×0.8–1.2	0.4–0.6×1.5–2.3	0.8–1.0×0.8–1.0	0.4–0.5×0.5–1.1	0.4–0.7×2.7–3.2
Cell shape	Rod	Rod	Rod	Rod	Cocci	Rod	Rod
**API 20NE**							
*β*-Glucosidase	+	+	+	nd	+	−	+
NO_3_^-^ to NO_2_^-^	+	+	nd	−	nd	−	−
Aesculin hydrolysis	+	+	+	+	+	−	+
**API ZYM**							
Oxidase	−	−	+	+	−	−	+
*β*-Galactosidase	+	+	−	−	−	+	+
Esterase	+	+	+	nd	nd	+	+
Lipase	−	−	+	−	nd	−	−
*α*-Chymotrypsin	+	+	+	w	nd	−	+
**Biolog GEN III**							
d-Cellobiose	+	+	nd	+	−	nd	−
d-Glucose	−	−	−	−	+	+	nd
d-Mannitol	−	−	nd	+	−	−	nd
Glycerol	−	−	nd	nd	+	nd	−
l-Rhamnose	−	−	nd	w	−	nd	−
Sucrose	−	−	nd	nd	+	nd	nd
l-Arabinose	−	−	nd	−	−	−	nd
Citrate	−	−	nd	nd	−	−	−
myo-Inositol	−	−	nd	nd	−	nd	nd
**Chemotaxonomic characteristics**							
Fatty acids	Summed feature 3 and summed feature 8	Summed feature 3 and summed feature 8	Summed feature 8, C_17 : 1_* ω6*c, and C_16 : 0_	C_18 : 1_*ω*7c, C_17 : 1_*ω*6c and summed feature 3	anteiso-C_15 : 0_, iso-C_14 : 0_ and iso-C_15 : 0_	C_18 : 1_*ω*7*c* and C_17 : 1_*ω*6*c*	Summed feature 8 and iso-C_18 : 0_
Quinone	Q-10	Q-10	Q-10	Q-10	Q-10	Q-10	Q-10, Q-9
Major polar lipids	DPG, PG, PC, PME, PL	DPG, PG, PC, PME, PL	PC, PE, DPG, PG, PL	SGL, PG, DPG, PE, PC, AGL, GL, PL	PG, GL, PC, PL	DPG, PE, PG	PG, PE, AGL, PL
G+C content (%)	61.23	61.39	62.71	60.80	71.50	66.40	61.60
Isolation source	Lake sediment	Lake sediment	Tidal flat sediment	Overlying water	Deep-sea sediment	Tidal flat	Intertidal surface seawater

Note: +, positive reaction; −, negative reaction; w, weakly positive; nd, not data available. Summed feature 3: C_16 : 1_* ω7*c and/or C_16 : 1_* ω*6*c*; summed feature 8: C_18 : 1_* ω*6*c* and/or C_18 : 1_* ω7*c.

Data were obtained using API 20NE, API ZYM and Biolog GEN III systems.

DPG, diphosphatidylglycerol; PC, phosphatidylcholine; PG, phosphatidylglycerol; PL, unidentified phospholipids; PME, phosphatidylmethylethanolamine.

### Chemotaxonomic analysis

The polar lipid profiles of two strains were composed of diphosphatidylglycerol, phosphatidylglycerol, phosphatidylmethylethanolamine, phosphatidylcholine and two unidentified phospholipids (Fig. S6). The major fatty acids (>10%) of strains EGI L300112^T^ and EGI L300120 were identified as summed feature 3 (C_16 : 1_* ω7*c and/or C_16 : 1_* ω6*c) and summed feature 8 (C_18 : 1_* ω6*c and/or C_18 : 1_* ω7*c). The detailed profile of fatty acids is provided in Table 2. The predominant respiratory quinone is ubiquinone Q-10.

**Table 2.** Cellular fatty acid profiles of strain EGI L300112^T^, EGI L300120 and their closely related type strains of the genus *Altererythrobacter* 1, EGI L300112^T^; 2, EGI L300120; 3, *A. litoralis* 1XM1-14^T^ [36] 4, *P. rhizovicinus* Ery12^T^ [37].

Fatty acid	1	2	3	4
**Straighted**				
C_11 : 0_	–	–	–	–
C_12 : 0_	tr	tr	0.6	
C_14 : 0_	tr	0.5	1.9	–
C_16 : 0_	5.5	10.8	**12.0**	4.4
C_17 : 0_	tr	tr	1.5	–
C_18 : 0_	tr	tr	tr	–
**Branched**				
iso C_10 : 0_	tr	tr	–	–
iso C_15 : 0_	1.1	1.0	–	–
iso C_16 : 0_	–	–	–	–
iso C_17 : 0_	–	–	–	–
iso C_18 : 0_	–	–	–	–
anteiso C_14 : 0_	1.5	5.5	–	–
anteiso C_15 : 0_	–	–	–	–
10-methyl C_19 : 0_	–	–	–	–
**Hydroxy**				
C_14 : 0_ 2-OH	5.1	5.3	3.2	9.6
C_16 : 0_ 2-OH	1.0	1.2	tr	tr
C_18 : 0_ 3-OH	–	–	–	–
C_18 : 1_ 2-OH	tr	tr	–	–
**Unsaturated**				
C_15 : 1_* ω*6c	tr	tr	–	–
C_16 : 1_* ω*5*c*	0.8	0.7	1.3	tr
C_17 : 1_* ω*8c	tr	tr	1.2	–
C_17 : 1_* ω*6c	5.3	5.0	21.7	1.3
C_18 : 1_* ω*5c	1.2	1.2	0.5	1.7
C_18 : 3_* ω*6c	tr	tr	–	–
C_20 : 1_* ω*7*c*	–	–	–	–
C_20 : 1_* ω*9*c*	–	–	–	–
C_19 : 0_ cyclo *ω*8*c*	0.7	2.8	tr	–
11-methyl C_18 : 1_* ω*7*c*	–	–	1.0	–
**Summed feature**				
3	18.0	16.0	9.4	5.6
8	54.9	45.4	30.1	72.8
9	1.1	1.4	0.6	–

tr, Trace (<0.5 %); –, not detected. Summed feature 3, C_16:1_ *ω7c* and/or C_16:1_ *ω6c*; summed feature 8; summed feature 9. Fatty acids amounting to more than 5% are highlighted in bold.

## Conclusion

The morphological, chemotaxonomic, phylogenetic and phylogenomic analyses consistently demonstrate that strains EGI L300112ᵀ and EGI L300120 represent a novel species within the genus *Altererythrobacter*. The two strains form a distinct lineage within the genus *Altererythrobacter* based on 16S rRNA gene sequence analysis and phylogenomic inference. In addition, the ANI and AAI values between the two strains and the closest related species of the genus *Altererythrobacter* are below the species-level thresholds, supporting their classification as a novel species of this genus. Furthermore, strains EGI L300112ᵀ and EGI L300120 can be clearly differentiated from related species of the genus *Altererythrobacter* by a combination of phenotypic, physiological and chemotaxonomic characteristics (Table 1). Based on the polyphasic taxonomic evidence, strains EGI L300112ᵀ and EGI L300120 are proposed to represent a novel species of the genus *Altererythrobacter*, for which the name *Altererythrobacter chaiwopuensis* sp. nov. is proposed.

## Description of *Altererythrobacter chaiwopuensis* sp. nov.

*Altererythrobacter chaiwopuensis* (chai.wo.pu.en’sis. N.L. masc. adj. *chaiwopuensis* of or belonging to Chaiwopu lake, a lake in Urumqi city, north-west China, where the strain was isolated).

Cells are aerobic, Gram-stain negative, non-motile and rod-shaped (0.4–0.6 µm×0.9–1.5 µm). Colonies on marine agar 2216 are yellow, circular and convex. Growth occurs at 15–37 ℃ (optimum, 28 ℃), pH 6.0–8.0 (optimum, pH 7.0) and with 0–7.0% (w/v) NaCl (optimum, 2.0 %, w/v). Catalase-positive and oxidase-negative. Tweens 20, 60 and 80 are hydrolysed, while H₂S production is negative. In API 20 NE tests, *β*-glucosidase and *β*-galactosidase activities are positive. API ZYM assays show activities of esterase (C4), esterase lipase (C8), leucine arylamidase, valine arylamidase, cystine arylamidase, trypsin, chymotrypsin, acid phosphatase, naphthol-AS-BI-phosphohydrolase and *α*-glucosidase. The sole respiratory quinone is ubiquinone Q-10. The major polar lipids are diphosphatidylglycerol, phosphatidylglycerol, phosphatidylmethylethanolamine, phosphatidylcholine and two unidentified phospholipids. The major fatty acids (>10 %) are summed feature 3 (C_16 : 1_* ω7*c and/or C_16 : 1_* ω6*c) and summed feature 8 (C_18 : 1_* ω6*c and/or C_18 : 1_* ω7*c). The DNA G+C content of the type strain is 61.2% based on the genome.

The type strain is EGI L300112ᵀ (=MCCC 1K09432ᵀ=KCTC 8712ᵀ), isolated from sediment of Chaiwopu Lake, Xinjiang, China. The GenBank accession numbers for the 16S rRNA gene sequence and draft genome sequence of strain EGI L300112^T^ are PX884856 and JBTXJF000000000, respectively.

## Supplementary material

10.1099/ijsem.0.007250 Supplementary Material 1.

## Abbreviations

AAI: amino acid sequence identity
ANI: average nucleotide identity
dDDH: digital DNA–DNA hybridization
PME: phosphatidylmethylethanolamine
