# Supplementary material for: Altererythrobacter chaiwopuensis sp. nov., a novel alphaproteobacterial strain isolated from sediment of the Chaiwopu Lake
Source: Int J Syst Evol Microbiol. 2026 Aug 10;76(8):007250. doi: 10.1099/ijsem.0.007250 (PMC13460513; doi:10.1099/ijsem.0.007250)
Supplement: Supplementary file 1 [file ijsem-76-07250-s001.pdf]

Supplementary Materials for IJSEM

Altererythrobacter chaiwopuensis sp. nov., a novel alphaproteobacterial strain isolated from sediment of the Chaiwopu Lake

Xin-Yao Li[1,5], Yi-Fei Yang[1], Yi-Wen Liang[4], Ze-Xian Wu[4], Shuai Li[1,2], Xu-Rui Li[1,2], Yong-Hong Liu[1,2], Lei Gao[1,2,3], Ting-Ting She[4], Bao-Zhu Fang[1,2]*, Wen-Jun Li[1,2,3]*

[1]State Key Laboratory of Ecological Safety and Sustainable Development in Arid Lands, China-Tajikistan Belt and Road Joint Laboratory on Biodiversity Conservation and Sustainable Use, Xinjiang Institute of Ecology and Geography, Chinese Academy of Sciences, Urumqi, 830011, PR China

[2]Xinjiang Key Laboratory of Biodiversity Conservation and Application in Arid Lands, Xinjiang Institute of Ecology and Geography, Chinese Academy of Sciences, Urumqi, 830011, PR China

[3]State Key Laboratory of Biocontrol, Guangdong Provincial Key Laboratory of Plant Stress Biology, School of Life Sciences, Sun Yat-Sen University, Guangzhou, 510275, Guangdong, PR China

[4]School of Biology and Food Engineering, Guangdong University of Education, Guangzhou, 510303， Guangdong, PR China

[5]University of Chinese Academy of Sciences, Beijing 100049, PR China

[*]Authors for correspondence:

Bao-Zhu Fang

E-mail: fangbaozhu@ms.xjb.ac.cn

Wen-Jun Li

E-mail: liwenjun3@mail.sysu.edu.cn

Table S1. Numbers of genes associated with the general COG functional categories.
1. EGI L300112^T; 2. EGI L300120; 3. *Altererythrobacter litoralis* 1XM1-14^T (Liu et al. 2024); 4. *Pelagerythrobacter rhizovicinus* Ery12^T (Gao et al. 2019).

Code	1	2	3	4
B	5	5	5	6
C	106	105	104	112
D	18	18	16	21
E	131	134	138	138
F	46	46	46	43
G	55	56	49	92
H	81	83	82	78
I	174	165	167	180
J	172	173	171	168
K	86	86	70	101
L	85	88	82	101
M	117	125	106	131
N	3	3	3	23
O	103	107	97	104
P	103	95	76	108
Q	37	36	62	37
R	99	97	89	121
S	65	67	68	90
T	76	77	66	88
U	25	26	25	34
V	33	35	44	47
W	13	13	13	6
Z	1	1	1	1

Note: B, Chromatin structure and dynamics; C, Energy production and conversion; D, Cell cycle control, cell division, chromosome partitioning; E, Amino acid transport and metabolism; F, Nucleotide transport and metabolism; G, Carbohydrate transport and metabolism; H, Coenzyme transport and metabolism; I, Lipid transport and metabolism; J, Translation, ribosomal structure and biogenesis; K, Transcription; L, Replication, recombination and repair; M, Cell wall/membrane/envelope biogenesis; N, Cell motility; O, Posttranslational modification, protein turnover; P, Inorganic ion transport and metabolism; Q, Secondary metabolites biosynthesis, transport and catabolism; R, General function prediction only; S, Function unknown; T, Signal transduction mechanisms; U, Intracellular trafficking, secretion, and vesicular transport; V, Defense mechanisms; W, Extracellular structures; Z, Cytoskeleton.

Table S2. Numbers of genes associated with the KEGG functional pathways.
1. EGI L300112^{T}; 2. EGI L300120; 3. *Altererythrobacter litoralis* 1XM1-14^{T} (Liu et al. 2024); 4. *Pelagerythrobacter rhizovicinus* Ery12^{T} (Gao et al. 2019).

KEGG_level2_pathway_ID	1	2	3	4
Amino acid metabolism	312	305	319	313
Biosynthesis of other secondary metabolites	31	35	29	37
Carbohydrate metabolism	329	328	308	365
Cell growth and death	39	39	37	45
Cell motility	10	9	3	50
Cellular commiunity	1	1	0	0
Energy metabolism	162	163	154	159
Enzyme families	0	0	0	1
Folding, sorting and degradation	42	42	41	40
Glycan biosynthesis and metabolism	33	31	26	30
Lipid metabolism	124	120	114	129
Membrane transport	52	53	52	62
Metabolism of cofactors and vitamins	135	139	139	129
Metabolism of other amino acids	74	74	78	81
Metabolism of terpenoids and polyketides	52	52	52	52
Nucleotide metabolism	90	90	87	89
Overview	297	297	326	297
Replication and repair	66	65	66	73
Signal transduction	97	97	80	108
Signaling molecules and interaction	1	2	2	1
Transcription	5	5	5	5
Translation	86	86	82	82
Transport and catabolism	19	19	15	19
Xenobiotics biodegradation and metabolism	124	110	180	105

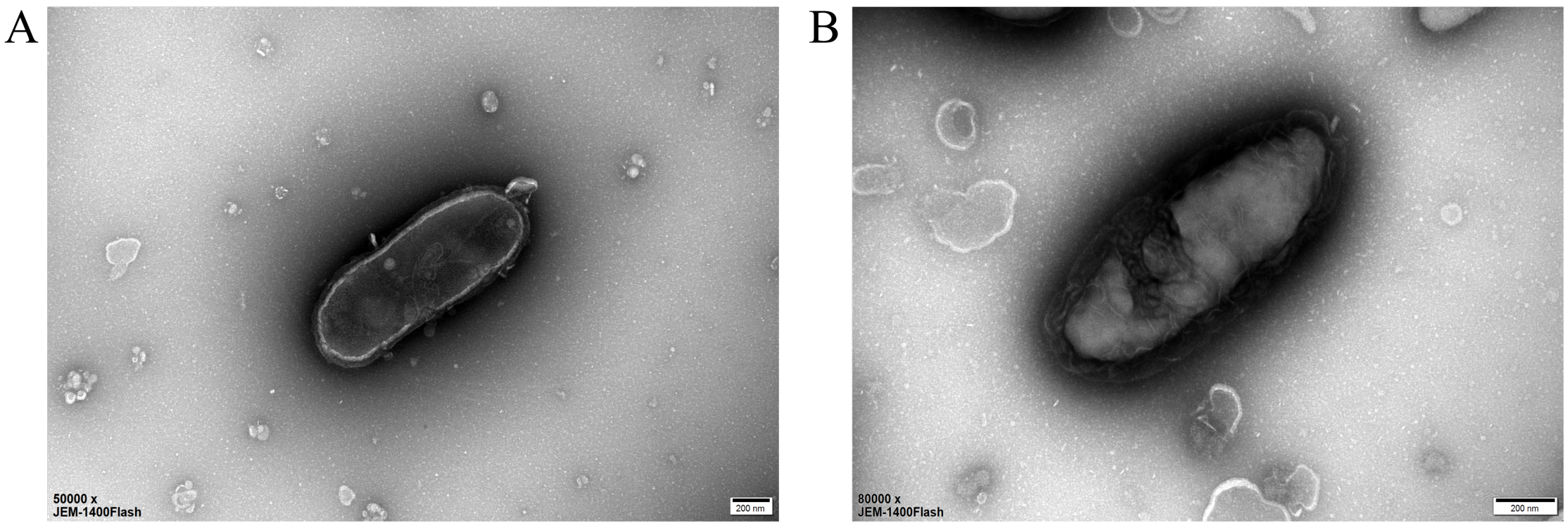

Fig. S1. Transmission electron microscopy image of strain EGI L300112^T (A) and EGI L300120 (B) grown on the marine agar 2216 at 30 ºC for 7 days. Bars, 200 nm.

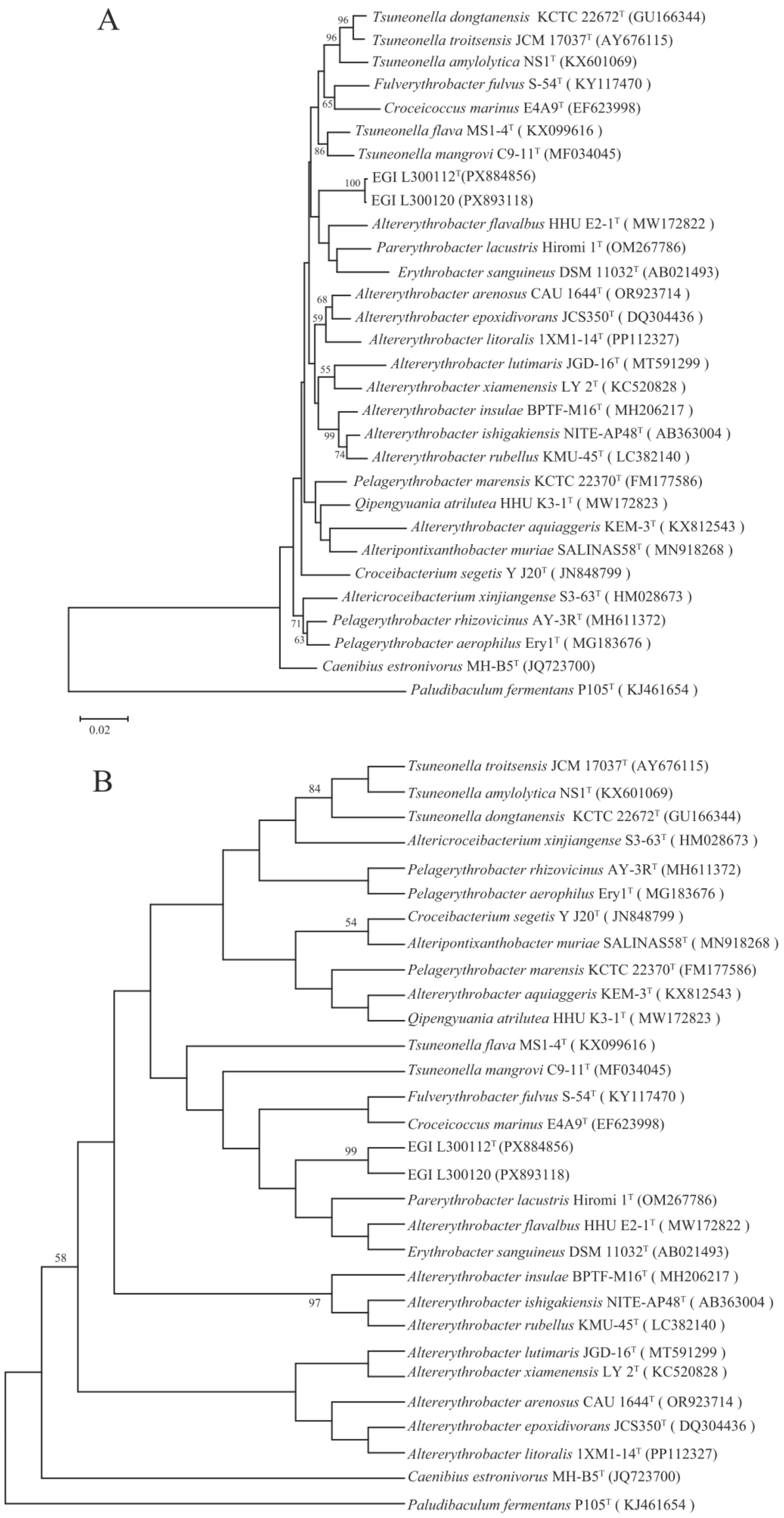

Fig. S2. Phylogenetic trees based on 16S rRNA gene sequences showing the phylogenetic relationships of strains EGI L300112^{T} and EGI L300120 within type strains of *Altererythrobacter* species and representatives of related taxa. (A) Neighbour-joining tree. (B) Maximum-parsimony tree. Bootstrap values (>50 %) based on 1000 resamplings are shown at branch nodes. *Paludibaculum fermentans* P105^{T} (KJ461654) was used as the outgroup. Bar, 0.05 substitutions per nucleotide position.

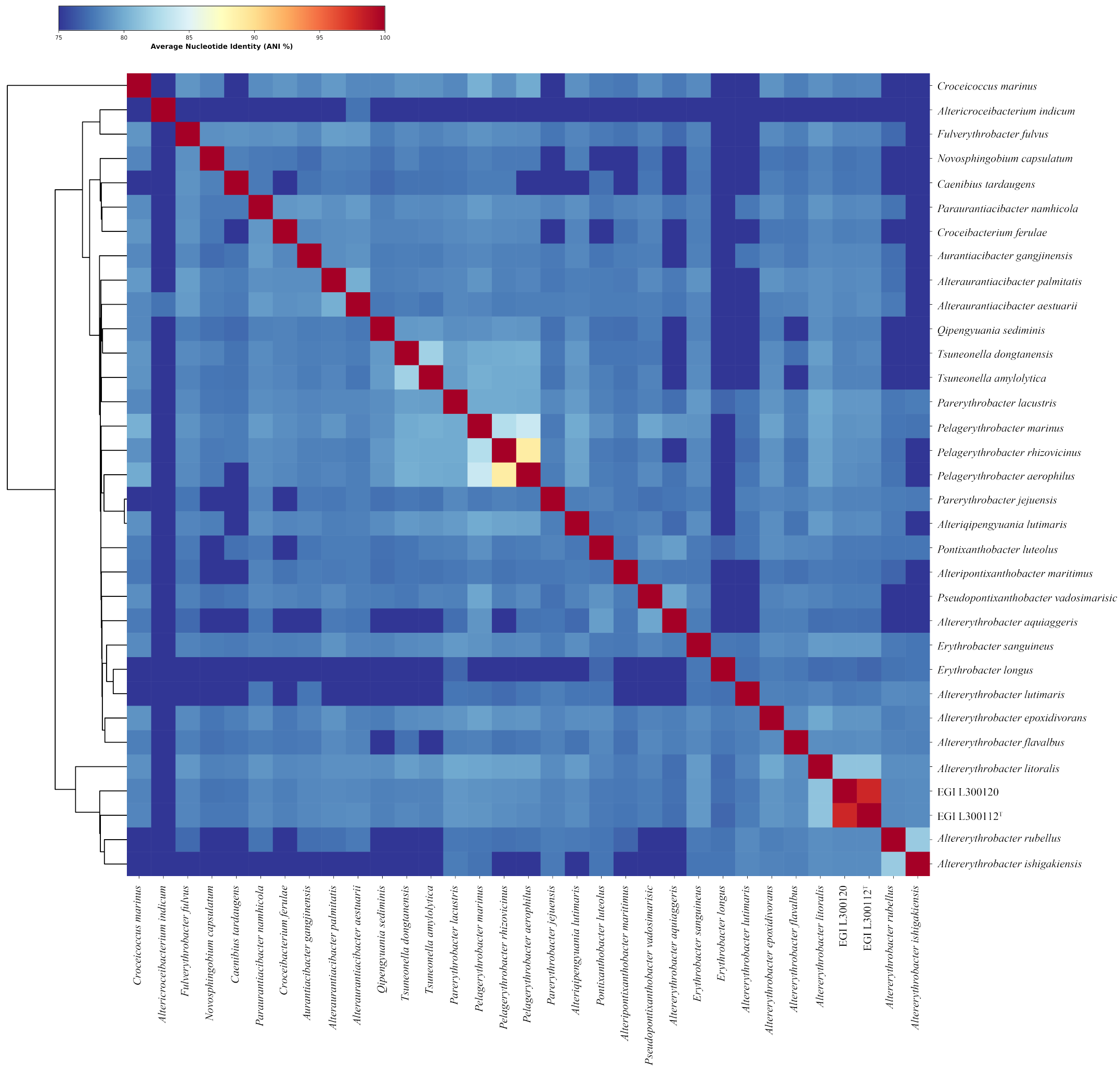

Fig. S3. Average Nucleotide Identity at genomic level of strain EGI L300112^T and EGI L300120 and closely related strains.

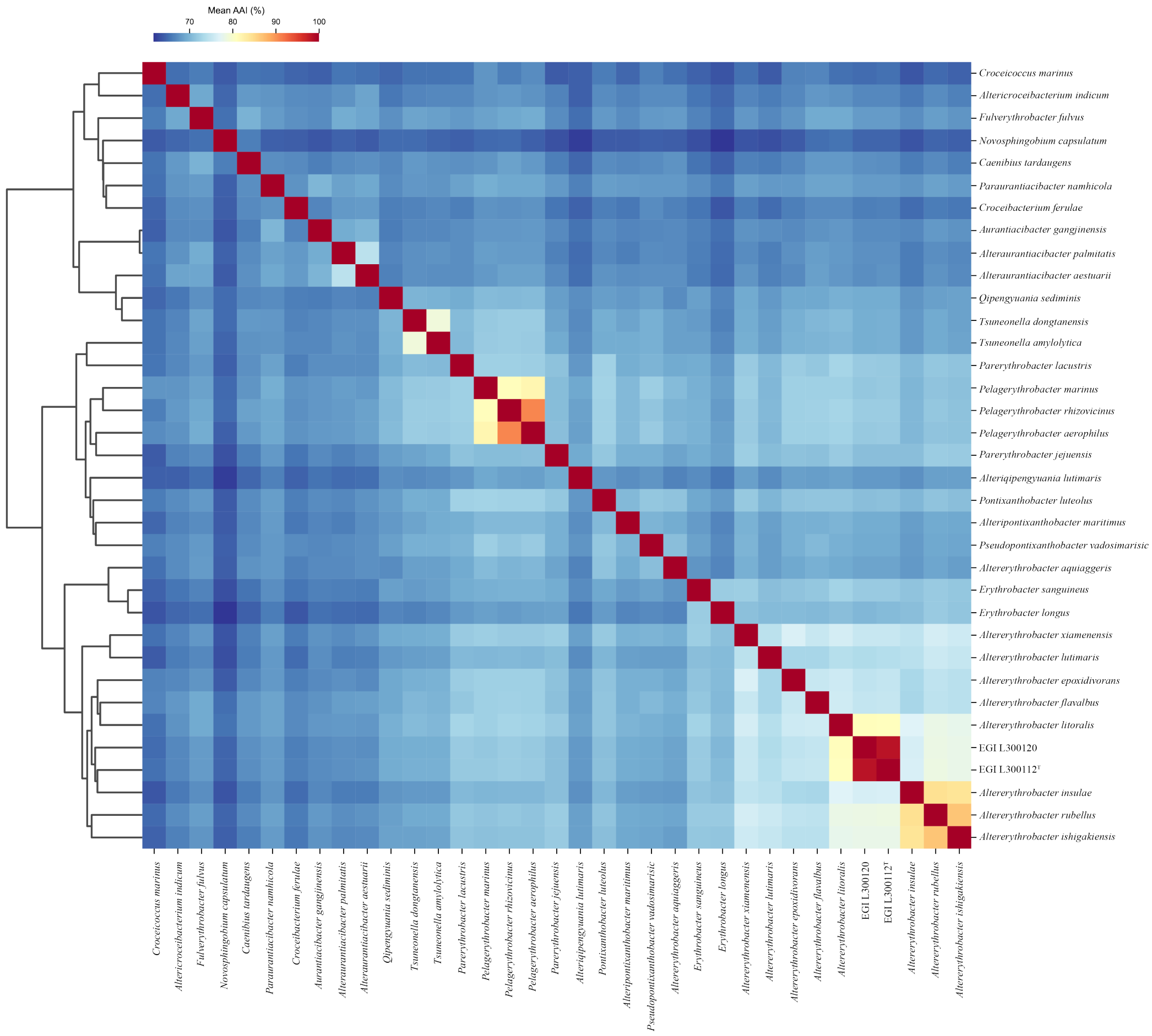

Fig. S4. Average amino-acid Identity at genomic level of strain EGI L300112^{T} and EGI L300120 and closely related strains.

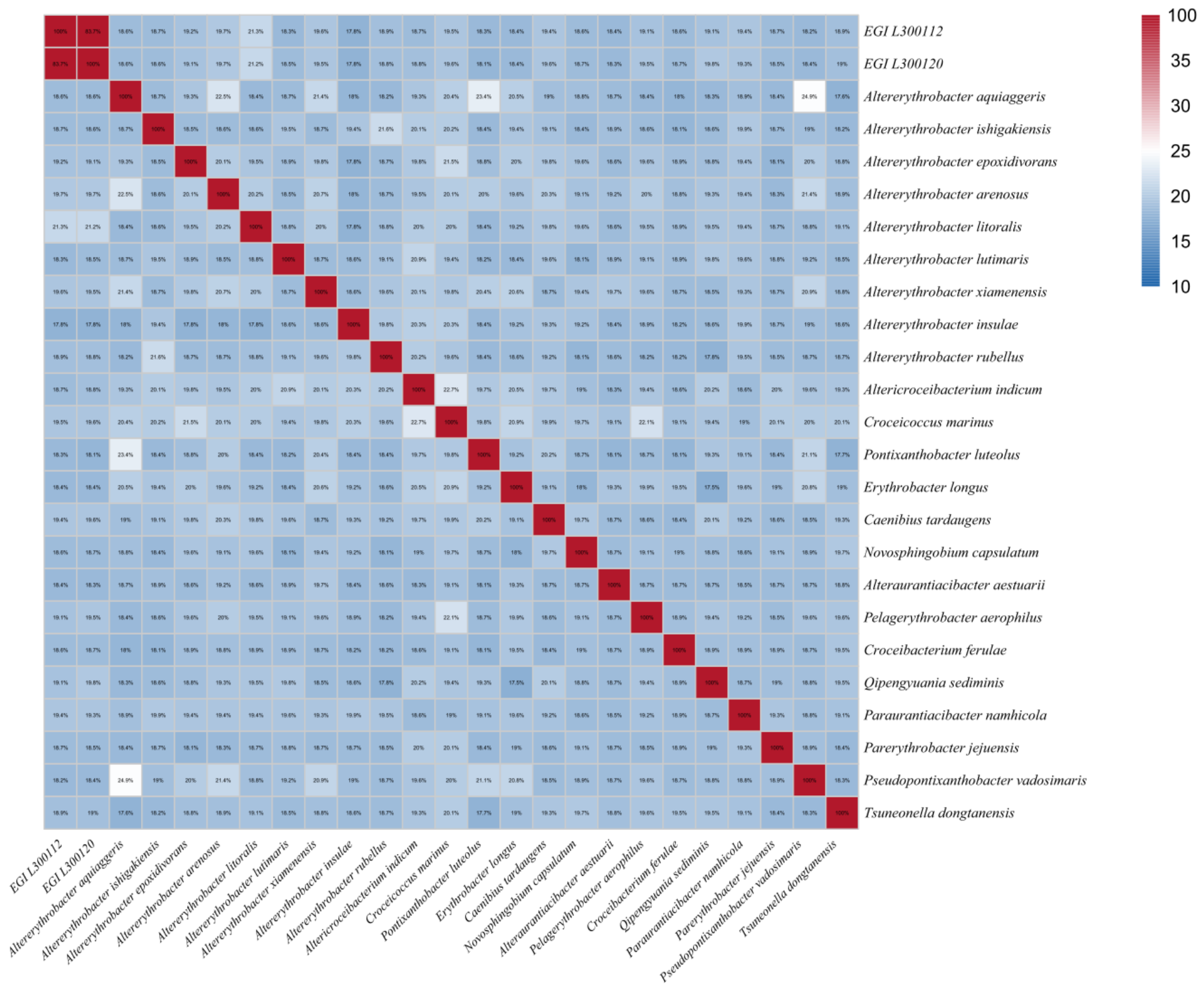

Fig. S5. Digital DNA–DNA hybridization (dDDH) values at the genomic level of strains EGI L300112^{T} and EGI L300120 and closely related strains.

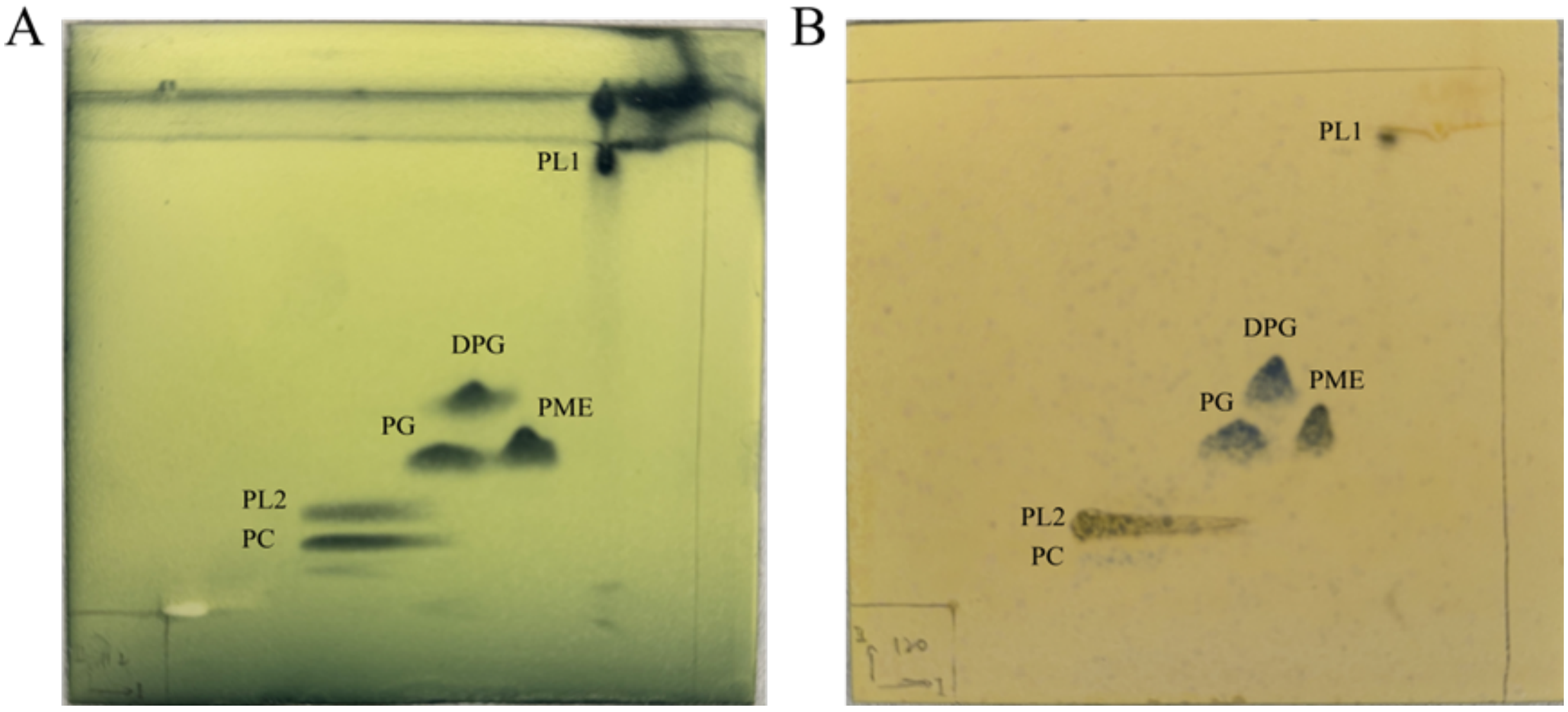

Fig. S6. Two-dimensional thin-layer chromatograms of polar lipids of strain EGI L300112^T (A) and EGI L300120 (B) following staining with 5 % ethanolic molybdophosphoric acid.

The chromatographic conditions were as follows: Silica Gel 60 thin-layer plates (10 by 10 cm) were spotted with 15 μL of a whole-cell lipid extract. Chloroform-methanol-water (65:25:4, vol/vol/vol) was used to develop the chromatogram in the first direction, and chloroform-acetic acid-methanol-water (80:18:12:5, vol/vol/vol/vol) was used in the second direction.

Abbreviations: DPG: diphosphatidylglycerol, PC: phosphatidylcholine, PME: phosphatidylmethylethanolamine, PG: phosphatidylglycerol, PL: unidentified phospholipids.
